# Lymphoscintigraphic alterations in lower limbs in women with lipedema in comparison to women with overweight/obesity

**DOI:** 10.3389/fphys.2023.1099555

**Published:** 2023-04-10

**Authors:** Angelika Chachaj, Ilona Dudka, Małgorzata Jeziorek, Monika Sowicz, Agnieszka Adaszyńska, Andrzej Szuba

**Affiliations:** ^1^ Department of Angiology, Hypertension and Diabetology, Wroclaw Medical University, Wroclaw, Poland; ^2^ Department of Chemistry, Umeå University, Umeå, Sweden; ^3^ Department of Dietetics and Bromatology, Faculty of Pharmacy, Wroclaw Medical University, Wroclaw, Poland

**Keywords:** lipedema, overweight, obesity, body mass index (BMI), lymphatic system, lymphoscintigraphy

## Abstract

**Introduction:** Lipedema is a bilateral enlargement of the legs due to abnormal depositions of subcutaneous fat. Recent studies using lymphoscintigraphy documented that lipedema associates with lymphatic alterations. It is still not known, whether non-lipedema obesity also leads to similar lymphoscintigraphic changes within lower legs. Clinically, both, lipedema and obesity may progress to secondary lymphedema. The aim of the study was to evaluate lymphoscintigraphy of lower limbs in women with lipedema in comparison to overweight/obese women.

**Methods:** 51 women (in the mean age of 43.3 ± 13.56) with the diagnosis of lipedema and 31 women (in the mean age of 44.7 ± 13.48) with overweight/obesity were enrolled into the study. Women in both study groups had no clinical signs of lymphedema. The groups were matched by mean volume of their legs, calculated using the formula for a truncated cone. Lymphoscintigraphy was evaluated in every women qualitatively. Body composition parameters were assessed using bioelectric impedance analysis (BIA).

**Results:** Lymphoscintigraphic alterations within lower extremities were similar in both, lipedema and overweight/obese groups and were present in majority of women in both study groups. The most common lymphoscintigraphic alteration in both groups were additional lymphatic vessels (in the lipedema group observed in 76.5% of patients and in the overweight/obesity group – in 93.5%). Visualization of popliteal lymph nodes and dermal backflow were observed respectively in 33% and in 5.9% in the group with lipedema and in 45.2% and in 9.7% in the overweight/obesity group. There were significant relationships between severity of lymphoscintigraphic alterations and weight, lean body mass (LBM), total body water (TBW), volume of both legs and thigh circumference in the lipedema group. Such relationships were absent in the overweight/obesity group.

**Discussion:** Our study indicates that lymphatic alterations are present before development to clinically visible secondary lymphedema in both conditions, lipedema and overweight/obesity. In majority of women from both study groups they indicate rather an overload of the lymphatic system than insufficiency. Lymphoscintigraphic alterations are similar in both groups, therefore, lymphoscintigraphy is not a diagnostic tool that might distinguish lipedema from overweight/obesity.

## Introduction

Lipedema is a chronic, progressive disorder, characterized by bilateral and symmetrical**,** pathological deposition of subcutaneous adipose tissue within the lower limbs and in one-third of cases also within upper limbs. Marked disproportion in size between the larger lower body and the smaller upper body is the first noticeable clinical symptom (Wold et al., 1951).

Lipedema affects women almost exclusively and may be recognized in even 11% of them (Fonder et al., 2007). The onset of lipedema most often occurs after rapid weight gain or during the period of hormonal changes (in adolescence, during and after pregnancy, less often during menopause) (Kruppa et al., 2020) (Herbst et al., 2021). The etiology of this condition is still unknown and many factors seems to be important, including genetic predisposition (Child et al., 2010) (Bano et al., 2010) (Michelini et al., 2020). Majority of patients report family history of lipedema (Child et al., 2010).

Women with this condition suffer from pressure-induced or spontaneous pain of adipose tissue and complain about the tendency to easy bruising even with minimal trauma (Wold et al., 1951). Characteristically, the feet are spared and there is a negative Stemmer’s sign (Vignes, 2012) (Warren et al., 2007). Secondary lymphedema occurs in the most advanced stage of this condition and is named lipolymphedema (Kruppa et al., 2020) (Keith et al., 2021). Other, less common signs are: skin hypothermia, hypermobile joints, tendency to developing telangiectasias and orthostatic edema (Herbst, 2012).

It was documented that lipedema is associated with impaired lymphatic transport in the studies using lymphoscintigraphy (Forner-Cordero et al., 2018) (Harwood et al., 1996) (Boursier et al., 2004) (Bilancini et al., 1995) (Gould et al., 2020) (Tartaglione et al., 2020), indocyanine green (ICG) lymphography (Zaleska et al., 2022) (Rasmussen et al., 2022) (Buso et al., 2022) and magnetic resonance (MR) lymphography (Lohrmann et al., 2009). It seems, that the more advanced stages of lipedema, the more inefficient the lymphatic system (Gould et al., 2020).

Lymphoscitigraphy is a diagnostic tool which is easy available to physicians diagnosing and treating patients with lipedema. Therefore, many often it might be the first and the only method used in evaluation of lymphatic system in this group of patients in daily medical practice (Forner-Cordero et al., 2018) (Herbst et al., 2021). However, it is still not known, whether non-lipedema obesity also leads to similar changes within lymphatic system in lower legs. Such suspicion is justified by the fact that obesity may lead to developing obesity-induced lymphedema (Mehrara and Greene, 2014) (Greene and Sudduth, 2021) (Sudduth and Greene, 2022).

The aim of the study was to evaluate the lymphoscintigraphic alterations in women with lipedema in comparison to overweight or obese women. Taking into account that patients with lipedema have elevated baseline BMI due to their abnormally enlarged lower extremities, we decided to match the study groups based on mean volume of their lower limbs. Characterization of all patients included also bioelectric impedance analysis.

## Material and methods

### Patients

51 women from Outpatient Angiology Clinic (with the mean age of 43.3 ± 13.56) with the diagnosis of lipedema without progression to lipolymphedema and 31 overweight/obese women (with the mean age of 44.7 ± 13.48) and without clinical signs of lipedema or obesity-induced lymphedema were enrolled into the study. The study groups were matched by age and mean volume of their lower limbs.

Diagnosis of lipedema in our patients was stated according to the criteria established by Wold et al. (1951). The minimal inclusion criteria to fulfill for the group with lipedema were disproportion in adipose tissue distribution within lower legs in comparison to upper body, negative Stemmer’s sign and presence of at least one of two clinical signs, i.e.,: 1) spontaneous pain or pain on pressure and/or 2) tendency to easy bruising.

The exclusion criteria for both groups were as follows: lymphedema, edema in the course of chronic vein insufficiency, heart failure, renal and hepatic insufficiency, abnormal TSH, cancer, pregnancy, period of at least 6 months after pregnancy and diabetes.

Lipedema group was classified into 3 clinical stages of severity and into 5 types in accordance with the localization of the inappropriate accumulation of fat (Kruppa et al., 2020). Table 1 presents the criteria used in our study to classify the women with lipedema.

**TABLE 1:** Criteria of stages and types of lipedema used in the study.

Lipedema - stages	Lipedema- types
1: the skin surface is normal	1: buttocks
2: the skin surface is irregular, pitted and orange skin phenomenon is present	2: buttocks, hips and thighs
3: deformation of legs with larger fat masses	3: buttocks, hips, thighs and calves
	4: lower extremities and arms
	5: calves

Ethical approval was provided by the Local Bioethical Committee of the Wroclaw Medical University (KB-690/2017). All women gave informed written consent prior to inclusion in the study following the principles outlined in the Declaration of Helsinki.

### Lymphoscintigraphy

Injections of 0.25 mCi of 99mTc-Nanocoll were given simultaneously in both foot in the second and the third interdigital space (Szuba and Rockson, 1998) (Szuba et al., 2003). Static whole body images were acquired 15 min and 2 h after the injection. Lymphoscintigraphy was evaluated in every women qualitatively by independent estimation by two physicians. Any difference in estimation was discussed and consensus was reached. The following parameters were assessed:

1) minor alterations:
  - asymmetry in visualization of inguinal lymph nodes
  - additional lymphatic vessels (collateral circulation)
2) moderate alterations:
  - visualization of popliteal lymph nodes (meaning significant lymphatic flow through the deep lymphatic system)
  - dermal backflow
3) severe alterations:
  - lack of visualization of inguinal lymph nodes.

We assigned/scored one point for the presence of each minor alteration, 2 points for each moderate alteration and 3 points for severe alteration. In order to estimate the severity of lymphatic system insufficiency, the sum of counted points was calculated and was named “lymphoscintigraphy value” in further analysis.

### Measurement of anthropometric measurements

Leg volume was calculated from leg circumferences measured at 4 cm distances using the formula for a truncated cone (Casley-Smith, 1994). Waist, hip, thighs (in the middle of the thighs), calves (in the middle of the calves) and ankles circumferences were measured with a standard tape measure to the nearest 1 cm. Body height [cm] was obtained by a TANITA HR- 001 growth meter (Tanita, Japan).

Waist-to-height ratio (WHtR) was calculated as the ratio of waist circumference [cm] to height [cm].

### Measurement of body composition parameters using BIA (bioelectric impedance analysis)

Parameters such as weight [kg], percentage body fat (PBF) [%], mass of body fat (MBF) [kg], lean body mass (LBM) [kg], total body water (TBW) [kg], and visceral fat level (VFL) were obtained by a TANITA MC-780MA 8-electrode body composition analyzer (Tanita, Japan). Patients were instructed not to consume food or drink for 4 h, not to engage in vigorous physical activity for 12 h, and not to use diuretics for 6 h prior to the study. Body Mass Index (BMI) was calculated as the ratio of body weight [kg] to height [m] squared.

### Statistical analysis

To compare clinical variables between studied groups Fisher’s exact test was used for categorical variables, Chi-square test for lymphoscintigraphy values and Student’s *t*-test for all other variables. Eta and Eta squared were used to evaluate the relationships between lymphoscintigraphy values (ordinal variable) and quantitative clinical parameters. Kendall’s tau-b correlation coefficient was used to evaluate the relationships between lymphoscintigraphy values (ordinal variable) and stage of lipedema (ordinal variable). *p*-values less than 0.05 were considered statistically significant. IBM SPSS Statistics software (version 29.0) and GraphPad Prism version 9.4.1 (GraphPad Software) were used for analyses.

## Results

### Patients clinical characteristics

The study groups did not differ in mean value of age, volume of their legs and circumferences at the level of hips, thighs, calves and ankles. The groups differed in weight, BMI, waist circumference, WHR, WHtR, MBF, and TBW, i.e., in the group with overweight/obesity these parameters were greater. Majority of women with lipedema reported a family history of this condition (78%). The onset of lipedema most often occurred during adolescence and pregnancy. In majority of women the first and second stage of lipedema (in total—88.2%) were assigned, the remaining women (11,8%) were in the third stage of the disease. The most frequent types of lipedema were type 2 (upper part of legs, in 43,1%) and 3 (whole legs, in 33,3%). Arms were affected in 23,5%. The most frequent clinical signs in women with lipedema were spontaneous or on pressure pain of adipose tissue (86,3%) and easy bruising (92,2%). Orthostatic oedema and teleangiectasias were less common (they were present in lipedema group in 68,6% and 43,1%, respectively). The frequency of these two last manifestations were statistically insignificant in comparison to overweight/obesity group. Selected medical parameters are summarized in Table 2.

**TABLE 2:** Clinical characteristics of subjects in groups with lipedema vs overweight/obesity.

Parameter	Lipedema group (*n* = 51)	Overweight/obesity group (*n* = 31)	*p-*values
Age (years) Mean ± SD	43.3 ± 13.56	44.7 ± 13.48	NS^a^
Age at onset	27.25 ± 14.70		
Family history (yes)	78%		
Onset during puberty (yes)	35.3%		
Onset/deterioration during pregnancy	29.4% (in the whole group)		
	42.6% (in the group being pregnant before)		
Onset/deterioration after pregnancy	9.8% (in the whole group)		
	14.3% (in the group being pregnant before)		
Onset/deterioration during menopause	9.8% (in the whole group)		
	14.3% (in the group it concerns)		
Stage of lipedema			
1	43.1%		
2	45.1%		
3	11.8%		
Type of lipedema			
1 (buttocks)	0%		
2 (buttocks, hips and thighs)	43.1%		
3 (from hips to ankles)	33.3%		
4 (arms and legs)	23.5%		
5 (calves)	0%		
Pain (spontaneous or on pressure) (yes)	86.3%	3.2%	<0.0001^b^
Easy bruising (yes)	92.2%	19.4%	<0.0001^b^
Orthostatic oedema (yes)	68.6%	51.6%	NS^b^
Telangiectasias (yes)	43.1%	41.9%	NS^b^
Stemmer’s sign (possitive)	0%	0%	
Volume of lower limbs [ml]			
left leg	13189 ± 3,258	12623 ± 3,748	NS^a^
right leg	13072 ± 3,414	12269 ± 3,360	NS^a^
mean volume of both legs	13130 ± 3,321	12446 ± 3,537	NS^a^
Hight	165.4 ± 7.402	166.1 ± 6.532	NS^a^
Weight	86.53 ± 20.99	99.65 ± 21.47	0.0080^a^
BMI [kg/m2]	31.8 ± 8.187	36.0 ± 7.192	0.0200^a^
normal	25.5%	0%	
overweight	17.6%	22.6%	
1st class obesity	27.5%	29.0%	
2nd class obesity	15.7%	25.8%	
3rd class obesity	11.7%	16.1%	
extreme obesity (>50 kg/m^2^)	2.0%	6.5%	
Arterial hypertension (yes)	23.5%	22.6%	NS^b^
Insuline resistance (yes)	23.5%	32.3%	NS^b^
Metformin taking (yes)	11.8%	3.2%	NS^b^
Treated hypothyroidism (yes)	23.5%	32.3%	NS^b^
Waist circumference	97.3 ± 16.41	111.3 ± 14.19	0.0002^a^
Hip circumference	115.3 ± 13.93	119.2 ± 13.90	NS^a^
WHR (waist-hip ratio)	0.8440 ± 0.09181	0.9339 ± 0.06227	<0.0001^a^
WHtR (waist-to-hight ratio)	0.5902 ± 0.1096	0.6705 ± 0.08550	0.0008^a^
Left thigh circumference [cm]	65.41 ± 8.411	67.45 ± 8.003	NS^a^
Right thigh circumference [cm]	65.36 ± 8.136	67.65 ± 7.975	NS^a^
Left calf circumference [cm]	44.33 ± 5.188	43.77 ± 5.128	NS^a^
Righ calf circumference [cm]	44.30 ± 5.186	43.76 ± 5.096	NS^a^
Left ankle circumference [cm]	25.18 ± 3.039	24.45 ± 3.012	NS^a^
Right ankle circumference [cm]	24.95 ± 2.938	24.50 ± 3.011	NS^a^
LBM (lean body mass) [kg]	52.1 ± 8.681	56.0 ± 9.096	NS^a^
PBF (percentage body fat) [%]	37.9 ± 7.043	40.5 ± 4.909	NS^a^
MBF (mass of body fat) [kg]	33.9 ± 113.21	40.61 ± 13.22	0.0304^a^
TBW (total body water) [kg]	38.6 ± 6.475	41.6 ± 6.688	0.0476^a^
VFL (visceral fat level)	12.6 ± 5.015	11.1 ± 4.023	NS^a^

Legend: Data presented as percentage (%) or mean ± standard deviation.

^a^ Student’s *t*-test.

^b^ Fisher’s exact test.

^c^ Chi-square test

NS, not statistically significant.

### Lymphoscintigraphic evaluations

**FIGURE 1:**
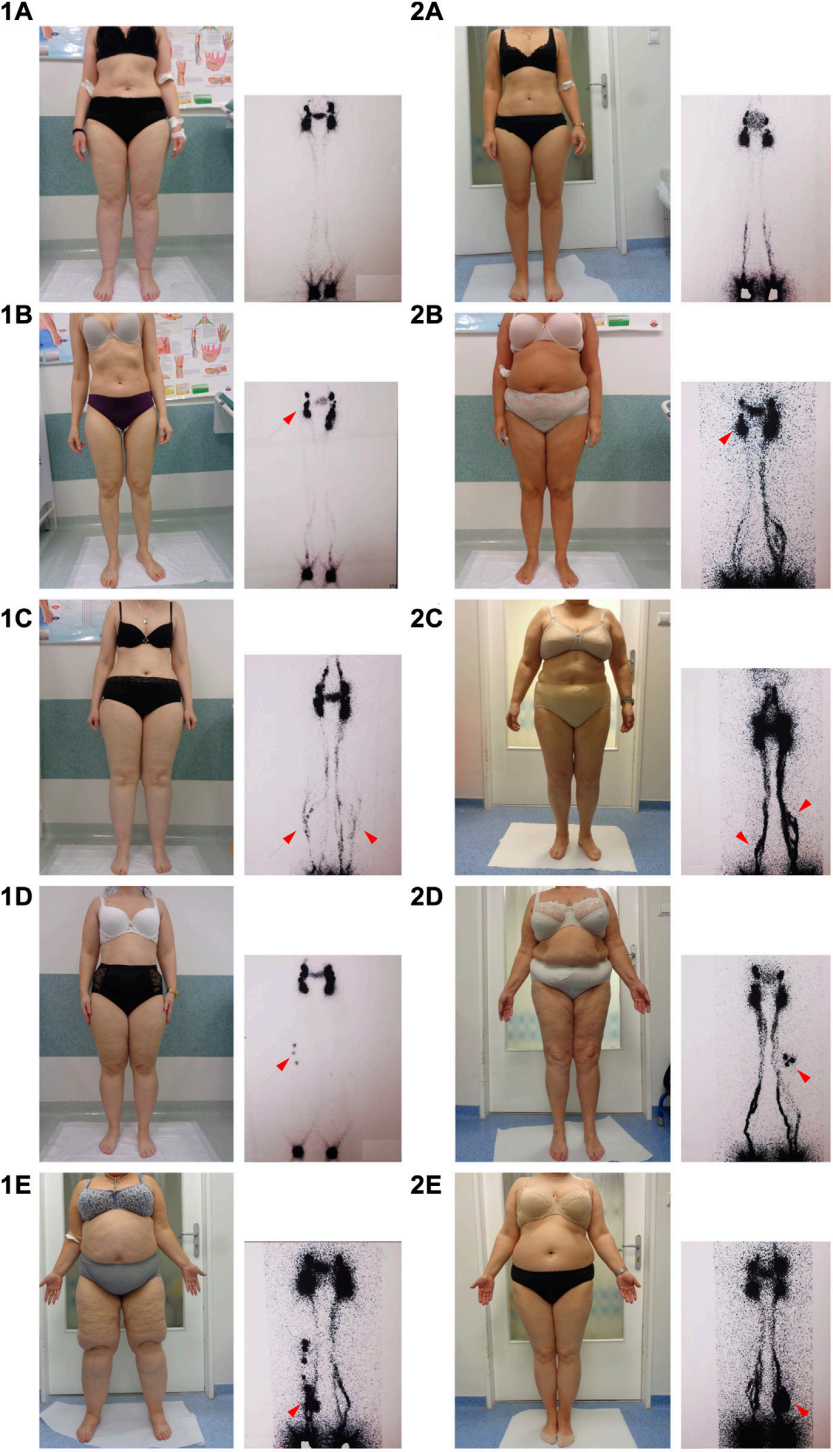
Examples of lymphoscintigraphic alterations in the group with lipedema and in the group with overweight/obesity. Arrows indicate sequential lymphoscintigraphic alterations listed in order of severity. However, several lymphoscitigraphic alterations were often observed in one patient. Legend: **(1A–E)**: lymphoscintigraphy in women with lipedema; **(2A–E)**: lymphoscintigraphy in women with oveweight/obesity; **(1A,2A)**: normal lymphoscintigraphy; **(1B,2B)**: asymmetry in visualization of inguinal lymph nodes; **(1C,2C)**: additional lymphatic vessels (collateral circulation); **(1D,2D)**: visualization of popliteal lymph nodes; **(1E,2E)**: dermal backflow.

**FIGURE 2:**
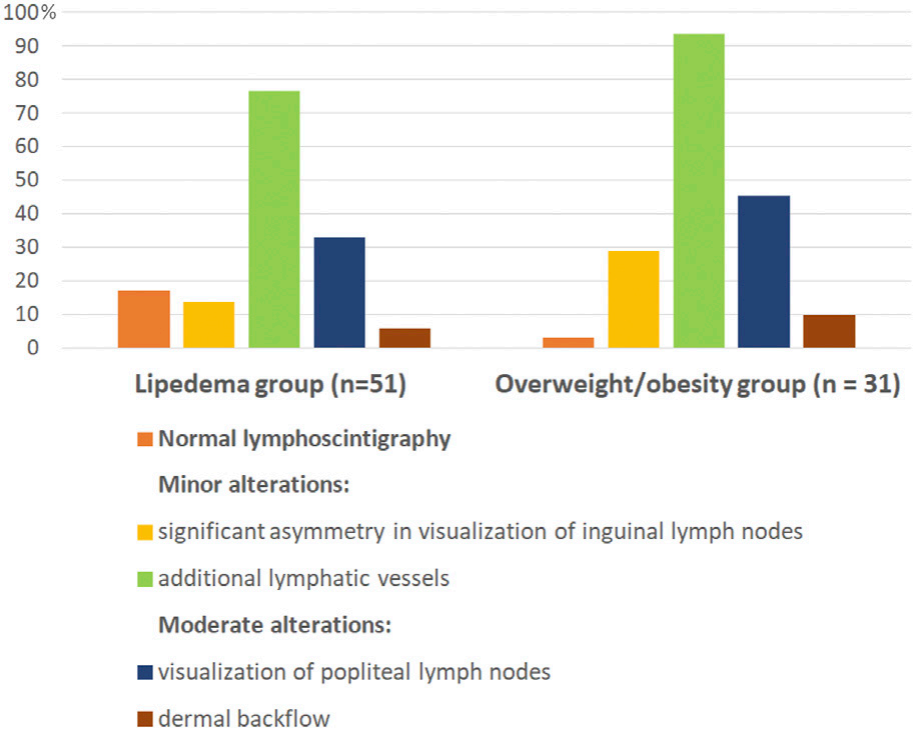
Graphically presented lymphoscintigraphic alterations in the study groups. Lipedema and overweight/obesity group did not differ in the type of lymphoscitigraphic alterations. Differences in percentages of individual lymphoscitigraphic alterations between the two groups were not statistically significant. All lymphoscitigraphic alterations in both study groups were of minor or moderate grade.

Majority of women in both study groups had features of lymphatic alterations. Normal lymphoscintigraphy was noted only in 17% in the lipedema group and in 3.2% in the overweight/obesity group. Comparison of lymphoscintigraphic evaluation of lower limbs between the study groups showed no statistically significant differences in both, severity and type of lymphatic alterations. The examples of alterations in lymphoscintigraphy of women from both study groups are presented in Figure 1. The summary of qualitative analysis of lymphoscintigraphic alterations in the study groups is presented in Table 3; Figure 2.

**TABLE 3:** Lymphoscintigraphic alterations in groups with lipedema vs. overweight/obesity.

Lymphoscintigraphic features	Lipedema group (*n* = 51)	Overweight/obesity group (*n* = 31)	*p*-value
Normal lymphoscintigraphy	17%	3,2%	**NS** ^a^
Minor alterations (1 point for each)	76,5%	93,5%	NS^a^
- significant asymmetry in visualization of inguinal lymph nodes	13,7%	29%	NS^a^
- additional lymphatic vessels	76,5%	93,5%	NS^a^
Moderate alterations (2 points for each)	37,3%	51,6%	NS^a^
- visualization of popliteal lymph nodes	33%	45.2%	NS^a^
- dermal backflow	5,9%	9,7%	NS^a^
Severe alterations			
- lack of visualization of inguinal lymph nodes	0%	0%	
Overall mean score of lymphoscintigraphic value	1.686 ± 1.304	2.323 ± 1.222	NS^b^

^a^ Fisher’s exact test.

^b^ Chi-square test.

Minor lymphoscintigraphic alterations were observed in majority of women in both groups (in 76.5% in the lipedema group and in 93.5% in the overweight/obesity group), and visualization of additional lymphatic vessels (collateral vessels) was the most common sign in both groups (Table 3; Figures 1C, 2C). Significant asymmetry in visualization of inguinal lymph nodes were less common (Table 3; Figures 1B, 2B). Moderate lymphatic alterations were observed in 37.3% in the lipedema group and in 51.6% in the overweight/obesity group and such result was largely due to the frequency of popliteal lymph node visualization (Table 3; Figures 1D, 2D). Dermal backflow was present in 5.9% in the lipedema group and in 9.7% in the overweigh/obesity group (Table 3; Figures 1E, 2E). Severe alterations (lack of visualization of inguinal lymph nodes) were absent in both groups.

Lymphoscintigraphic alterations were present in all stages and types of lipedema, also in women with normal BMI. The relationship between lymphoscintigraphic value and age in both groups was insignificant. The relations between lymphoscintigraphic value and stage of lipedema or BMI in both groups were also statistically irrelevant. In turn, there were statistically significant relationships between lymphoscintigraphic value and weight, LBM, TBW, volume of both legs and thigh circumference in the lipedema group. Such relationships were not significant in the overweight/obesity group.

**FIGURE 3:**
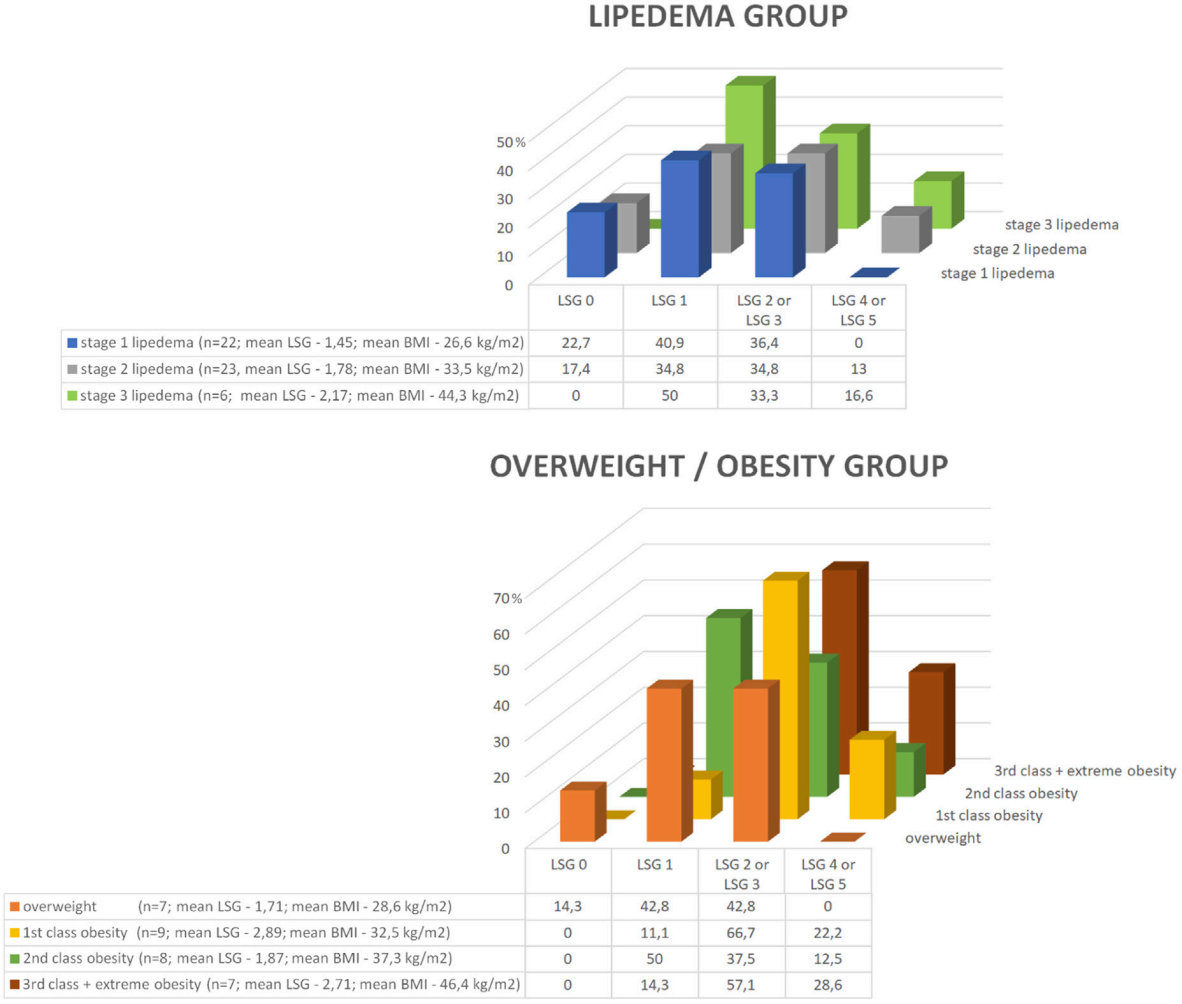
Graphically presented lymphoscintigraphic values in relation to stage of lipedema in the lipedema group or the degree of obesity in the overweight/obesity group. A trend indicating a relationship between stage of lipedema/degree of obesity and lymphoscintigraphic value can be noticed. However, the number of patients in the subgroups divided according to stage of lipedema or degree of obesity was too small for statistically significant results. Legend: BMI, body mass index; LSG, lymphoscintigraphic value.

However, when the lymphoscintigraphic values in relation to stage of lipedema in the lipedema group or degree of obesity in the overweight/obesity group are visually analyzed, a trend indicating a relationship between stage of lipedema/degree of obesity and lymphoscintigraphic value can be noticed (Figure 3). Nevertheless, the number of patients in the subgroups divided according to stage of lipedema or degree of obesity was too small for statistically significant results.

The summary of relationships between the lymphoscintigraphy values and clinical parameters in both study groups are showed in Table 4.

**TABLE 4:** The relationship between the lymphoscintigraphy values and other clinical parameters in the study groups.

Relationship between lymphoscintigraphy value and	Lipedema group (*n* = 51)			Overweight/obesity group (*n* = 31)		
	Eta (η)	Eta squared (η^2^)	*p-*value	Eta (η)	Eta squared (η^2^)	*p-*value
Age [years]	0.454	0.206	0.057	0.331	0.109	0.690
Volume of the left leg [ml]	0.475	0.226	0.036*	0.211	0.044	0.944
Volume of the right leg [ml]	0.543	0.295	0.006*	0.183	0.033	0.970
Mean volume of both legs [ml]	0.512	0.262	0.15	0.194	0.038	0.961
Hight [cm]	0.400	0.160	0.150	0.396	0.157	0.477
Weight [kg]	0.499	0.249	0.021*	0.399	0.159	0.470
BMI [kg/m^2^]	0.434	0.188	0.084	0.456	0.208	0.291
Waist circumference [cm]	0.399	0.159	0.154	0.410	0.168	0.434
Hip circumference [cm]	0.423	0.179	0.103	0.359	0.129	0.600
WHR (waist-hip ratio)	0.312	0.097	0.447	0.372	0.139	0.558
WHtR (waist-to-hight ratio)	0.279	0.078	0.584	0.390	0.152	0.499
Left thigh circumference [cm]	0.455	0.207	0.056	0.219	0.048	0.935
Right thigh circumference [cm]	0.488	0.238	0.027*	0.290	0.084	0.802
Left calf circumference [cm]	0.376	0.141	0.216	0.358	0.128	0.605
Right calf circumference [cm]	0.395	0.156	0.163	0.225	0.051	0.927
Left ankle circumference [cm]	0.424	0.180	0.100	0.182	0.033	0.971
Right ankle circumference [cm]	0.445	0.198	0.068	0.224	0.050	0.928
LBM (lean body mass) [kg]	0.592	0.350	0.001*	0.411	0.169	0.428
PBF (percentage body fat) [%]	0.265	0.070	0.639	0.375	0.140	0.549
MBF (mass of body fat) [kg]	0.417	0.174	0.114	0.384	0.148	0.517
TBW (total body water) [kg]	0.533	0.284	0.008*	0.345	0.119	0.645
VFL (visceral fat level)	0.250	0.063	0.699	0.528	0.278	0.125

*- statistically significant.

## Discussion

The most important observations from our study are as follows. 1) Lymphoscintigraphic alterations within lower limbs were similar in the group with lipedema and in the group with overweight/obesity. 2) Lymphoscintigraphic alterations were present in majority of women in both study groups. 3) Minor lymphatic alterations were the most common, especially visualization of additional lymphatic vessels. 4) Moderate changes were also present and visualization of popliteal lymph nodes were the most common; dermal backflow was present only in several women in each group. 5) Severe lymphoscintigraphic alterations were absent in both study groups and it was in agreement with physical examination (we excluded from the study patients with clinical sign of lymphatic insufficiency). 6) Lymphoscintigraphic value was statistically related to weight, LBM, TBW, volume of legs and thigh circumference in the lipedema group. 7) Lymphoscintigraphic alterations were statistically not related to clinical stage of lipedema and BMI in both groups, however, visually, there may be noticed a trend indicating a relationship between stage of lipedema/degree of obesity and lymphoscintigraphic alterations.

To our knowledge, only one other study has examined lymphoscintigraphy in obesity (Greene and Sudduth, 2021), however, the cited study was conducted on the group of women with greater BMI and towards searching lymphoscintigraphic alterations indicative of secondary lymphedema. In our study, women with overweight and in all obesity classes were evaluated. Moreover, to our knowledge, for the first time the lipedema group was compared with another group in lymphoscintigraphic study after matching the groups by volume of legs. Such groups matching seems to be optimal in evaluating lymphoscintigraphy of lower extremities in the studies comparing lipedema patients with non-lipedema obesity patients.

The lymphoscintigraphic alterations found in our study in both groups indicated rather an overload, than insufficiency of lymphatic system. It is most evidenced by the visualization of additional lymphatic vessels and popliteal lymph nodes. Dermal backflow as a result of lymphatic insufficiency was a rare alteration in both groups in our study. In contrary, lymphoscitigraphic abnormalities in patients with lymphedema are often more prominent, including lack of visualization of inguinal lymph nodes and common presence of dermal backflow (Szuba et al., 2003).

Lymphoscintigraphic alterations in lipedema were reported also by other studies and, like in ours, they have shown that this condition is associated with abnormalities mostly of low or low-moderate grade (Forner-Cordero et al., 2018) (Harwood et al., 1996) (Boursier et al., 2004) (Bilancini et al., 1995) (Gould et al., 2020) (Tartaglione et al., 2020). In the study using rest/stress lymphoscintigraphy, the lymphoscintigraphic findings in 54 women with diagnose of lipedema were very similar to ours, i.e., it was documented that tortuous course of the lymphatic pathway and collateral flow were present in 75%, the presence of collaterals and/or popliteal node uptake in 49%, deep lymphatic vessels and popliteal node uptake in 36% and dermal backflow in 2.8% (Tartaglione et al., 2020). In other study with using dynamic lymphoscintigraphy, performed in 12 women with lipedema, beyond observation of abnormal lymphoscintigraphic pattern and slowing of the lymphatic flow, the attention has been drawn to asymmetry of lymphoscintigraphy disturbances, despite characteristic symmetric clinical involvement of legs in lipedema (Bilancini et al., 1995). ICG lymphography studies also revealed significant changes in superficial lymph flow in lipedema in comparison to control group, i.e.,: slower lymph flow, higher number of abnormal lymphatic vessels and higher fluorescent intensity in the skin (Zaleska et al., 2022), dilation of lymphatic vessels, intravascular pooling, and grater propulsion rates (Rasmussen et al., 2022). Lymphatic alterations estimated in ICG lymphography correlated with the duration of symptoms (Buso et al., 2022).

We did not find statistically significant relationships between lymphoscintigraphic alterations and BMI or age. These findings are in agreement with other study, in which they also demonstrated no relation of lymphoscintigraphic changes with BMI in lipedema (Forner-Cordero et al., 2018). However, in another lymphoscintigraphic study, it was documented that the risk of lymphedema in patients with lipedema or obesity may be predicted by BMI (Greene and Sudduth, 2021). In our study there was a significant relationship between severity of lymphoscintigraphic alterations and weight, LBM, TBW, volume of both legs and thigh circumference, but only in the lipedema group. We presume that the lack of similar relationships in overweight/obese women may be the result of heterogeneity of obesity, e.g., abdominal and gynoidal types of obesity. More research is needed in this regard.

## Limitations

Diagnosis of lipedema is based on physical examination and searching for positive diagnostic criteria of lipedema. There is still no reliable test to make a certain diagnosis of this condition. The distinction between lipedema and obesity is very difficult in some cases, especially in women with greater BMI, in which the disproportion of lower body to upper body is not so apparent due to greater amount of adipose tissue also within trunk. Therefore, we are aware that there was a possibility of some incorrect qualifications of women to the lipedema group vs. overweight/obesity group. However, we believe that the inclusion criteria used in our study minimized this possibility.

## Conclusion

Our study indicates that lymphatic alterations are present before development to clinically visible secondary lymphedema in both conditions, lipedema and overweight/obesity. Lymphoscintigraphic alterations in both study groups were of minor or moderate grade and indicated rather an overload, than insufficiency of lymphatic system. Lymphoscintigraphic alterations were similar in both groups, therefore, lymphoscintigraphy is not a diagnostic tool that might distinguish lipedema from overweight/obesity.

## Data Availability

The raw data supporting the conclusion of this article will be made available by the authors, without undue reservation.
